# Postoperative hydrocephalus in patients with infratentorial brain metastases may be influenced by preoperative treatment: a single-center cohort study

**DOI:** 10.1007/s11060-025-05125-7

**Published:** 2025-06-23

**Authors:** Luisa Mona Kraus, Maria Goldberg, Eugen Ursu, Kayra Demirbag, Simon Paul Backhaus, Ghaith Altawalbeh, Denise Bernhardt, Chiara Negwer, Stephanie Combs, Bernhard Meyer, Arthur Wagner

**Affiliations:** 1 Department of Neurosurgery, School of Medicine and Health, Klinikum Rechts Der Isar, University Hospital of TUM, Ismaninger Strasse 22, 81675 Munich, Germany; 2 Faculty of Medicine, Marmara University, Istanbul, Turkey; 3 Department of Radiation Oncology, TUM School of Medicine and Health, TUM University Hospital Rechts Der Isar, Technical University of Munich, Munich, Germany; 4 Helmholtz Zentrum München (HMGU), Institute of Radiation Medicine (IRM), Oberschleißheim, Germany; 5 German Consortium for Translational Cancer Research (DKTK), Partner Site Munich, Munich, Germany

**Keywords:** Infratentorial brain metastases, Hydrocephalus, Radiotherapy, Chemotherapy

## Abstract

**Purpose:**

Infratentorial brain metastases (BM), particularly those causing obstruction of the fourth ventricle, are associated with a significant risk of postoperative hydrocephalus. This complication remains poorly understood, especially regarding its predictors beyond mechanical obstruction. This study aims to identify clinical predictors of postoperative hydrocephalus in patients undergoing surgery for infratentorial BM.

**Methods:**

We performed a single-center retrospective analysis of 235 adult patients surgically treated for infratentorial BM between 2009 and 2025. Patients with leptomeningeal disease were excluded. Pre- and postoperative hydrocephalus were defined based on imaging and clinical criteria. Logistic regression and multivariate modeling were used to evaluate predictors, including clinical presentation, treatment history, and imaging features.

**Results:**

Postoperative hydrocephalus occurred in 18.45% of patients. Breast cancer patients exhibited the highest incidence (30.61%), significantly more than those with lung cancer (15.66%, p = 0.042). Preoperative hydrocephalus (p = 0.005), and prior chemotherapy (p = 0.001) or radiotherapy (p = 0.004) were significantly associated with postoperative hydrocephalus. Imaging variables, including tumor volume or proximity to the fourth ventricle, were not predictive. Multivariate regression confirmed preoperative hydrocephalus, and systemic treatment as independent risk factors.

**Conclusion:**

Postoperative hydrocephalus in infratentorial BM is influenced not only by mechanical factors but also by preoperative clinical and therapeutic variables. Breast cancer patients, particularly those who received prior systemic or local therapy, are at higher risk. These findings suggest the need for individualized risk assessment and raise the question of whether prophylactic interventions could mitigate complications and treatment delays in high-risk cohorts.

## Introduction

Brain metastases (BM) are the most common intracranial tumors, affecting 20–30% of cancer patients. They are to this day still associated with poor prognosis [1]. Lung and breast cancer are the most common entities to metastasize to the brain [2]. Gastrointestinal cancer (GIT) has recently shown an increased tendency to metastasize to the brain. Survival in these patients is diminished compared to our cancer entities like lung or breast cancer [3]. Postoperative treatment mainly consists of stereotactic radiotherapy of the cavity or whole brain radiotherapy in case of multimetastatic disease [4, 5]. In solitary brain metastases there is no overall survival benefit in supratentorial compared to infratentorial metastases [6].

Infratentorial location in general is associated with a higher postoperative complication rate [7]. Tumor-associated hydrocephalus is a serious and life-threatening complication.

Tumors obstructing the fourth ventricle and mechanically hindering cerebrospinal fluid (CSF) outflow have a higher incidence of hydrocephalus [8]. Known independent imaging predictors of postoperative hydrocephalus are tumor-volume-to-edema or fourth-ventricle-ratio suggesting that hydrocephalus in patients with infratentorial BM is a mechanical problem due to the confined space of the posterior fossa [9]. Other than the obstructive nature of infratentorial BM or the decreased CSF reuptake early works have named hypersecretion another source of hydrocephalus [8]. In brain cancer, postoperative hydrocephalus is also associated with operative complications such as CSF leak or infection [10]. The literature reports a mean incidence of hydrocephalus in infratentorial BM of approximately 20%. in adults [11].

Multifactorial, tumor-associated hydrocephalus may persist or develop only after surgery. While this complication is well known [12], management strategy has widely been discussed. This may, in part, be attributable to insufficient knowledge of clinically relevant factors. To tackle this disadvantage and improve decision making in patients with persisting postoperative hydrocephalus, we have performed a single-center, retrospective analyses aiming to identify clinical predictors.

## Methods

We performed a single-center, retrospective data bank analyses of all patients treated surgically for infratentorial brain metastases from 2009 to 2025.

The local ethics committee granted permission for this study (No.5626:12). Since a retrospective chart review was performed no additional patient consent was required.

Our inclusion criteria were:

- Diagnosis of a malignant tumor disease
- Age > 18 years
- Planned surgery for one or more intracerebral, infratentorial brain metastases
- Preoperative MRI

Our exclusion criteria were:

- Sole supratentorial or brainstem location
- Known history of leptomeningeal disease (LM) diagnosed on MRI or via lumbar puncture prior to index surgery

### Primary endpoints

Primary endpoints were preoperative and/or postoperative hydrocephalus in the acute/non-acute presentation setting.

Diagnosis of preoperative hydrocephalus was made on MRI as reported by a neuroradiologist.

Postoperative hydrocephalus was diagnosed when either (i) as acute on postoperative imaging requiring immediate drainage by external ventricular drainage (EVD) or as (ii) late onset requiring shunt implantation.

### Definition of independent variables

Acute presentation included all visits that were triggered by hydrocephalic OR cerebellar symptoms and that resulted in imaging showing infratentorial BM. Non-acute presentations included all visits that followed imaging diagnosis of infratentorial brain metastases with or without accompanying symptoms.

Preoperative hydrocephalus was objectively defined on preoperative MRI by the presence of triventricular dilation and periventricular CSF capping. Proximity to fourth ventricle was operationally defined as either (i) occlusion of such on MRI or (ii) tumor expansion into the fourth ventricle. Lesions confined to the parenchyma without involvement of the fourth ventricle were considered not to meet this criterion. Tumor volume was calculated using BrainLab Smart Brush Tool.

Only preoperative radiotherapy of the neurocranium, including obligatory radiosurgery of the index metastasis, was considered. No difference was made between stereotactic or whole brain radiation.

Any revision ranging from wound healing disorder, deep infection, CSF leakage or postoperative bleeding surgery was considered.

Complete resection was defined as no visible residual tumor on the first postoperative MRI during postoperative hospital stay.

### Statistical analyses

GraphPad Prism (version 10.4.2) and IBM SPSS (version 30.0.0) were used for statistical analyses and data interpretation. All data was assessed for Gaussian distribution. Medians or means were further used accordingly. Medians were compared using the Mann–Whitney-U test. Proportions were compared using the Fisher’s exact test.

Logistic regression was primarily used for prediction analyses. Alternatively, Firth’s logistic regression was used when perfect separation was detected. Log rank test was used for survival analyses. Multicollinearity was assessed by the Variance inflation factor (VIF) and assumed when VIF was > 10 or Tolerance was < 0.10. Significancy levels were * in p < 0.05 and ** in p < 0.005.

Deepnote was used for image illustration.

## Results

### Data exploration

From 2009 to 2025 we treated 235 patients with one or multiple infratentorial brain metastases by surgery. Sex was nearly equally distributed; 122 patients were female, 113 were male. Median age was 66 years (IQR 56–74). Cancer type was lung in 83 patients (35.32%), breast in 49 patients (20.85%), melanoma in 14 patients (5.96%), CUP in 5 patients (2.13%), GIT in 42 patients (17.87%), RCC in 8 patients (3.40%), urothelial carcinoma in 6 patients (2.55%) or others in 27 patients (11.49%). Rate of preoperative hydrocephalus was 34.62% and of persisting postoperative hydrocephalus was 18.38%.

Epidemiological, preoperative and postoperative clinical data as per cancer type are depicted in Table 1a–c.

**Table 1:** Epidemiological (**a**), preoperative (**b**) and postoperative (**c**), clinical data per cancer type

a	Cancer type	n	Age [years]	Sex [% females]	Acute presentation	Karnofsky [%]	Tumor volume [ml^3^]
	Lung	83	66.70 (58.00–75.00)	38.55%	69.88%	70	13.93 (7.16–21.83)
	Breast	49	60.02 (49.00–69.00)	100.00%	69.39%	80	14.45 (10.16–24.80)
	Melanoma	14	59.93 (46.00–71.00)	35.71%	35.71%	75	12.65 (3.58–35.24)
	CUP	5	72.40 (60–83.50)	40.00%	100.00%	60	13.6 (9.21–19.85)
	GIT	42	66.81 (58.75–74.00)	35.71%	78.57%	70	15.7 (10.30–21.20)
	RCC	8	63.38 (54.25–77.00)	37.50%	75.00%	85	9.67 (2.71–18.35)
	Urothelial	6	71.33 (64.00–80.25)	16.67%	66.67%	75	9.99 (5.54–21.21)
	others	27	65.59 (57.00–73.00)	55.56%	70.37%	70	18.74 (12.54–29.52)

b	Cancer type	n	Preoperative radiotherapy	Preoperative chemotherapy	Preoperative steroids	Hydrocephalus on preop. MRI	EVD preop
	Lung	83	15.66%	12.05%	26.51%	32.05%	6.02%
	Breast	49	14.29%	36.73%	34.69%	27.08%	10.20%
	Melanoma	14	15.38%	7.69%	7.14%	35.71%	14.29%
	CUP	5	0.00%	0.00%	20.00%	60.0%	0.00%
	GIT	42	9.52%	21.43%	28.57%	48.78%	4.76%
	RCC	8	12.50%	0.00%	50.00%	42.86%	12.5%
	Urothelial	6	16.67%	33.33%	50.00%	66.67%	0.00%
	others	27	3.70%	14.81%	14.81%	30.77%	3.70%

c	Cancer type	n	Postoperative Hydrocephalus	EVD postop	Shunt	Revision rate	Time to death [months]
	Lung	83	15.66%	8.43%	13.25%	15.66%	5 (1.00–15.00)
	Breast	49	30.61%	20.41%	20.40%	20.40%	6 (3–15.75)
	Melanoma	14	14.29%	14.29%	7.14%	28.57%	10 (2.00–26.00)
	CUP	5	0.00%	0.00%	0.00%	0.00%	23 (4.00–42.00)
	GIT	42	21.43%	4.76%	14.29%	11.90%	3 (2.50–13.50)
	RCC	8	14.29%	12.5%	0.00%	0.00%	12.5 (0.25–41.25)
	Urothelial	6	0.00%	0.00%	0.00%	33.33%	3.5 (2.00–5.00)
	others	27	11.11%	3.70%	11.11%	3.70%	7 (2.00–15.50)

Table shows medians for numeric variables and percentages for binary variables

Age is given in years, time from diagnosis to death is given in months

Revision rate included any kind of revision surgery

Looking for significant differences in epidemiological and clinical data of the cancer types we performed an ANOVA with post-hoc correction for age, tumor volume and time from diagnosis to death. While age was statistically significant (p = 0.0343) post-hoc correction showed none of the pairwise differences were large enough to reach significance after correction. There was no statistically significant difference in tumor volume or time from diagnosis to death.

Comparing all binary variables between cancer types we found almost no statistically significant differences after performing multiple Fisher’s exact tests with Bonferroni correction (Table 1). The only significant parameter was proportion of female sex as 100% of patients with breast cancer were female.

### Data analyses at primary endpoint

Postoperative hydrocephalus was reported in 43 of 235 patients (18.45%). The rate of postoperative hydrocephalus was highest in breast cancer (30.61%) and lowest in CUP and urothelial carcinoma in which no cases of postoperative hydrocephalus were reported (Table 1). Comparing breast cancer to the other cancer types in which postoperative hydrocephalus was found, there was a significant difference compared to most common type, lung cancer (15.66% vs. 30.61%; p = 0.042). Preoperative hydrocephalus did significantly correlate with postoperative hydrocephalus over all cancer types (p = 0.005) while acute presentation did also correlate with preoperative hydrocephalus (p = 0.014).

**Fig. 1:**
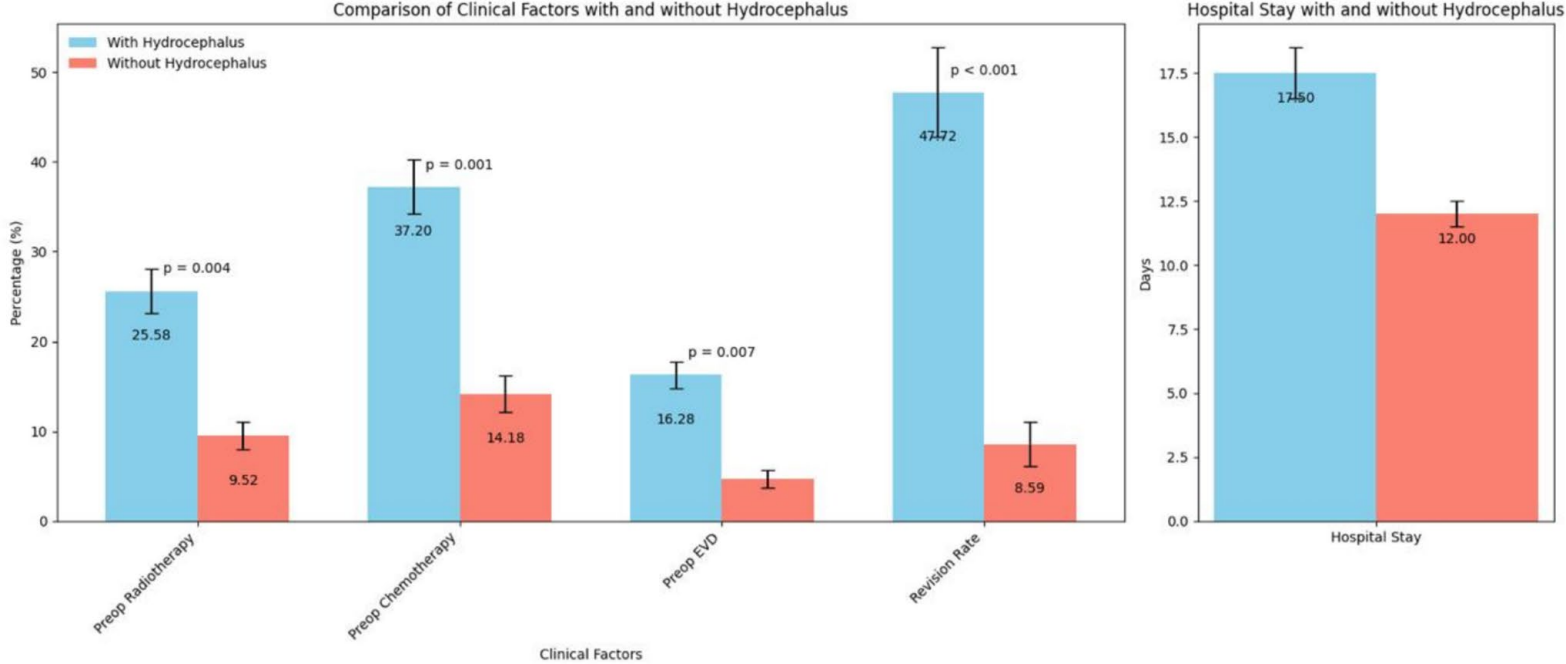
Comparison of relevant independent clinical variables in patients with and without postoperative hydrocephalus

Comparing patients with postoperative hydrocephalus to patients without postoperative hydrocephalus there was no significant difference in age (65.65 vs. 64.80 years; p = 0.687), tumor volume (12.39 ml^3^ vs. 15.12 ml^3^; p = 0.130), proportion of female sex (62.79% vs. 50.00%; p = 0.131) or rate of complete resection (69.77% vs. 85.55% (p = 0.564). Significant differences were found in patients treated with preoperative radiotherapy (25.58% vs. 9.52%; p = 0.004) or preoperative chemotherapy (37.20% vs. 14.18%; p = 0.001), and in patients with preoperative EVD (16.28% vs. 4.73%; p = 0.007). Furthermore, there was a significant difference in revision rate for patients with and patients without postoperative hydrocephalus (47.72% vs. 8.59%; p < 0.001) as well as in length of hospital stay (17.50 days vs. 12 days; p < 0.001) (Fig. 1). Revision surgery was performed following postoperative bleeding in 38.10%, posterior fossa swelling in 14.30%, infection in 19.04% and active CSF leakage in 28.57%.

Sub cohort analyses of patients with early onset hydrocephalus and late onset hydrocephalus without prior EVD placement revealed that there were no differences in age, proportion of female sex, tumor volume, preoperative radiotherapy, or preoperative chemotherapy.

### Survival analyses

**Fig. 2:**
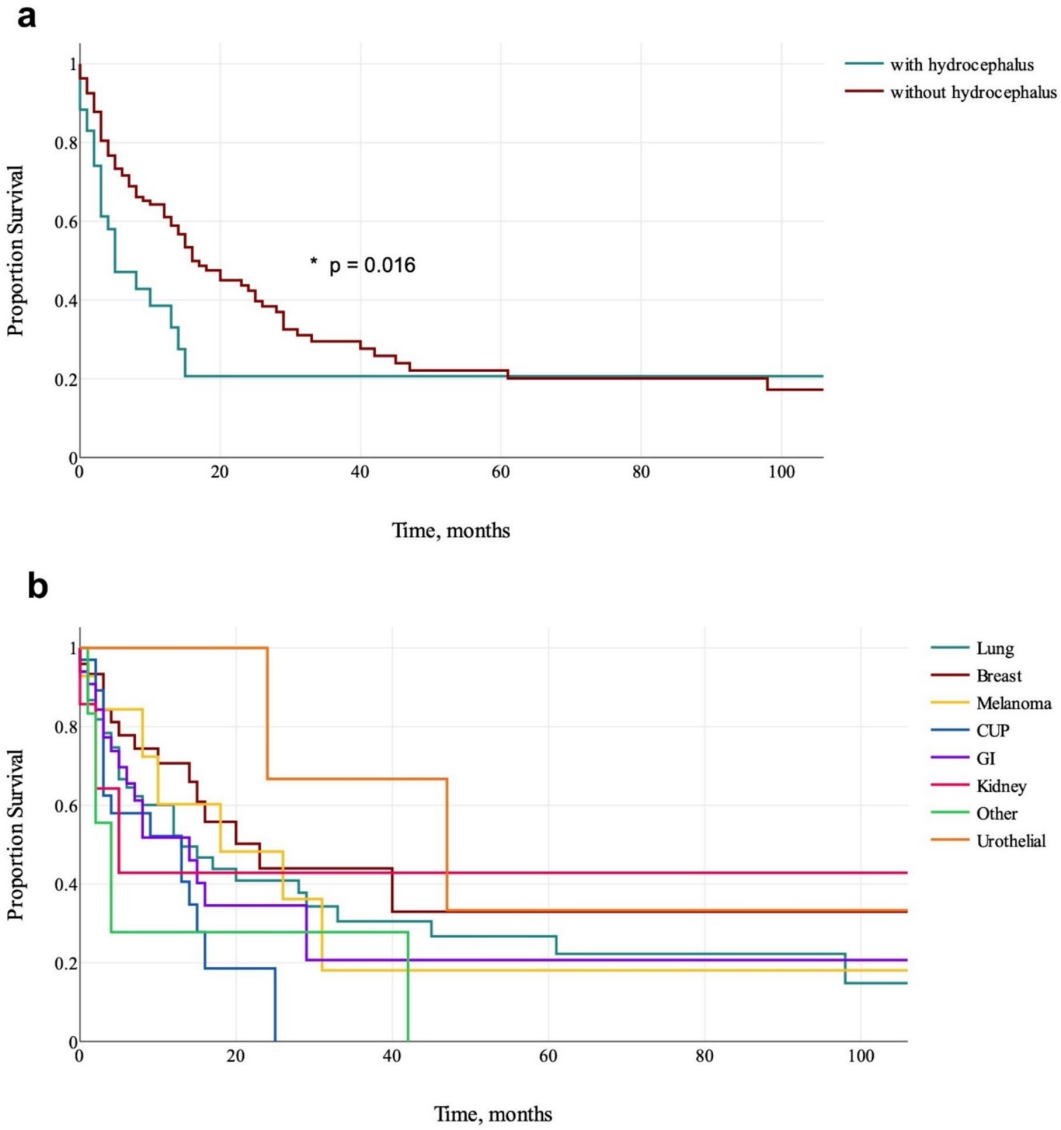
Kaplan–Meier curves of patients with and without postoperative hydrocephalus (**a**) and of all patients grouped by cancer type regardless of hydrocephalus diagnosis (**b**) Median survival time is given in months

Median survival in patients with postoperative hydrocephalus was 5 months (95% C.I. = 3–13) and 16 months (95% C.I. = 14–26) in patients without postoperative hydrocephalus. There was a significant difference (p = 0.016) (Fig. 2a).

Grouped by cancer type there was no significant difference in median survival (p = 0.130) (Fig. 2b). Median survival ranged from 4 months (95% C.I. = 2–4) in other cancer types to 47 months (95% C.I. = 24–47) in urothelial cancer.

### Prediction models

To look for independent predicting factors of postoperative hydrocephalus we first performed simple logistic regression analyses (Table 2). Statistically significant variables were preoperative hydrocephalus (p = 0.006), preoperative EVD (p = 0.011), preoperative radiotherapy (p = 0.006) and preoperative chemotherapy (p = 0.001).

**Table 2:** Simple regression analyses of relevant variables over all cancer types for data set exploration

			95% C.I. for OR	
	p-value	OR	Lower	Upper
Female sex	0.132	1.687	0.854	3.333
Age	0.685	1.006	0.979	1.033
Acute	0.258	0.670	0.335	1.341
Hydro preop	0.006*	2.609	1.318	5.164
EVD preop	0.011*	3.910	1.368	11.180
Volume	0.559	0.993	0.968	1.018
4th ventricle	0.740	1.238	0.351	4.364
Multiple	0.305	1.435	0.720	2.860
Radiotherapy	0.006*	3.266	1.410	7.562
Chemotherapy	0.001**	3.407	1.630	7.122
Dexamethasone	0.887	1.055	0.504	2.210
Time to death	0.016*	0.901	0.828	0.981
Complete resection	0.411	0.685	0.292	1.746

*acute* acute presentation, *hydro preop* Hydrocephalus on preoperative imaging, *EVD preop* EVD placement because of acute symptoms of hydrocephalus prior to surgery, *volume* overall infratentorial tumor volume, *4th ventricle* location of metastasis in proximity to the fourth ventricle, *multiple* multiple infratentorial brain metastases, *OR* odds ratio, *C.I.* confidence interval

Next, we performed multiple regression analyses strictly accounting for sex and age. Variables were grouped in clinical (Table 3a), therapeutic (Table 3b) and imaging variables (Table 3c). Regarding clinical variables at first presentation, we identified preoperative hydrocephalus (p = 0.013) and acute presentation (p = 0.031) as significant predictors. In this scenario sex was also statistically significant (p = 0.022).

**Table 3:** Multiple regression analyses of relevant variables over all cancer types grouped into clinical (**a**), therapeutic (**b**) and imaging variables (**c**)

			95% C.I. for OR	
	p-value	OR	lower	upper
a				
Female sex	0.022*	2.451	1.138	5.278
Age	0.158	1.022	0.992	1.053
Acute	0.031*	0.425	0.195	0.924
EVD preop	0.068	3.063	0.921	10.178
Hydro preop	0.013*	2.686	1.235	5.843
b				
Female sex	0.247	1.539	0.745	3.140
Age	0.139	1.022	0.993	1.053
Radiotherapy	0.032*	2.816	1.093	7.252
Chemotherapy	0.008**	2.992	1.329	6.740
Dexamethasone	0.460	0.734	0.323	1.667
c				
Female sex	0.335	1.812	0.541	6.603
Age	0.452	1.019	0.970	1.072
4th ventricle	0.645	1.402	0.330	5.987
Volume	0.809	1.004	0.975	1.033
Multiple	0.302	1.847	0.576	5.919
Complete resection	0.231	0.342	0.058	2.181

*acute* acute presentation, *hydro preop* Hydrocephalus on preoperative imaging, *EVD preop* EVD placement because of acute symptoms of hydrocephalus prior to surgery, *volume* overall infratentorial tumor volume, *4th ventricle* location of metastasis in proximity to the fourth ventricle, *multiple* multiple infratentorial brain metastases, *OR* odds ratio, *C.I.* confidence interval

Accounting for preoperative forms of cancer treatment radiotherapy (p = 0.032) and chemotherapy (p = 0.008) were found to be statistically significant predictors.

Imaging variables such as tumor volume, tumor location near the fourth ventricle or the occurrence of multiple infratentorial metastases were not found to be statistically significant predictors.

No evidence of multicollinearity was found among the independent variables, as all variance inflation factor (VIF) values were below 10 (Table 4).

**Table 4:** Multicollinearity analysis

	Tolerance	VIF
Female sex	0.86	1.16
Age	0.85	1.17
Acute	0.82	1.22
Hydro preop	0.64	1.57
EVD preop	0.73	1.38
Volume	0.88	1.13
4th ventricle	0.68	1.47
Multiple	0.93	1.07
Radiotherapy	0.78	1.28
Chemotherapy	0.76	1.31
Dexamethasone	0.87	1.15
Complete resection	0.94	1.06

## Discussion

The results of this study indicate that the development of postoperative hydrocephalus in infratentorial BM is not only influenced by the anatomical location but also by preoperative clinical factors. Our work has been looking beyond the common understanding that has widely believed postoperative hydrocephalus to be a volume problem of the limited posterior fossa.

Shabo et. al showed that the tumor-to-edema-ratio as well as the tumor-to-fourth ventricle-ratio were significant predictors of postoperative hydrocephalus while they could—in concordance with our findings—find no correlation of the absolute tumor volume at all [9]. These findings support the common hypothesis that postoperative hydrocephalus in patients with infratentorial BM is the result of a lack of space—especially if there is tumor edema or postoperative swelling. The perioperative usage of dexamethasone for anti-edematous therapy did not significantly influence the incidence of postoperative hydrocephalus in our cohort. Median tumor volume did not differ significantly either.

To estimate a prediction model of postoperative hydrocephalus in infratentorial BM, we analyzed relevant clinical factors. As expected, preoperative hydrocephalus showed a significant correlation with postoperative hydrocephalus. Interestingly, placement of an EVD in an emergency setting prior to surgery was associated with preoperative hydrocephalus but not postoperative hydrocephalus. Acute preoperative intervention in severe cases could have potentially led to decreased risk of postoperatively persisting hydrocephalus.

Comparing both outcomes, we found significant differences in the medial therapy that led up to surgery. There was a significant higher rate of patients that later suffered from postoperative hydrocephalus in patients that had had preoperative radiotherapy and/or chemotherapy. This does not seem to be related to a mass effect. Systemic chemotherapy and local radiotherapy of the brain are often combined in the treatment of metastatic disease. Our multicollinearity analyses could not show a strong correlation between radiotherapy and chemotherapy, however. They seem to increase the risk for postoperative hydrocephalus independently.

As a relevant rate of hydrocephalus persists after tumor removal or develops postoperatively alternative theories have been emerging. Complications leading to revision surgery were significantly more common in the hydrocephalus cohort suggesting that infection and bleeding may play a role in the development of postoperative hydrocephalus. In addition to occlusive hydrocephalus, clinicians also recognize communicating hydrocephalus resulting from impaired cerebrospinal fluid resorption. Obliteration of the arachnoid villi may cause CSF outflow restriction in post-hemorrhagic hydrocephalus. Similar mechanisms have been proposed in brain metastases [13]. Additionally, apart from diminished CSF egress, there could be an overproduction. Hydrocephalus in BM has only recently been proposed to result from a disturbed cellular architecture of the central nervous system [14, 15]. Macrophages regulate CSF flow and their genetic depletion results in CSF accumulation [16]. Mast cell migration to the choroid plexus promotes CSF production, and thus leads to non-mechanical hydrocephalus in BM [15]. Radiation triggers neuroinflammatory response and increases concentration of inflammatory markers in the CSF [17]. These inflammatory markers could disturb the delicate cellular environment. Our clinical data simultaneously identified preoperative radiotherapy as well as chemotherapy as predictors of postoperative hydrocephalus. The highest rate of preoperative chemotherapy was found in breast cancer (36.73%). Breast cancer was also the cancer type with the highest rate of postoperative hydrocephalus (30.61%). Every fifth patient with breast cancer in our cohort required shunting postoperatively. As overall survival and progression free survival in these patients is significantly influenced by adjuvant treatment radiotherapy should be started as soon as possible [5]. Additional surgeries may delay further treatment or interrupt systemic or radiotherapy that has already been initiated. Based on imaging characteristics scoring systems of postoperative hydrocephalus have been introduced [18]. Aligning with our data, preoperative hydrocephalus increases the probability of persisting postoperative hydrocephalus. Prophylactic shunting in infratentorial tumors has long been subject of discussion [12]. A recent metanalysis reports a persisting hydrocephalus rate of 13.63%. The mean rate over all cancer types in our cohort was similar with 18.38%. The rate in breast cancer, however, was still distinctly higher. The difference was statistically significant compared to the most common cancer type lung. Given that patients with breast cancer also exhibit the longer median survival, anticipating potential postoperative complications seems relevant. As hydrocephalus is also a consequence of LM, which may be particularly relevant in metastatic breast cancer [19], we deliberately excluded patients with LM in this analysis.

## Limitations

This study is limited by its monocentric nature and retrospective character. While preoperative radiotherapy was identified as an independent predicting factor of postoperative hydrocephalus, no differentiation on stereotactic versus whole brain radiation. Future insights on dosage and radiation exposure of the ventricular system could yield additional information. While we are aware that our retrospective data should be interpreted carefully, we tentatively propose that for a certain patient cohort there are predictors of persisting postoperative hydrocephalus other than imaging properties.

## Conclusion

Patients with infratentorial breast cancer metastases regardless of tumor size or location within the posterior fossa seem to be at higher risk for postoperative hydrocephalus requiring intervention. Those who have already received local radiotherapy and/or systemic treatment are more likely to be affected. In this selected patient cohort preemptive intervention could prove to be useful to reduce postoperative delay of further treatment. Based on our retrospective data further prospective analyses could be warranted. To our knowledge there is no prospective work evaluating the potential benefit of prophylactic shunting on multimodal treatment success.

## Data Availability

No datasets were generated or analysed during the current study.

## Abbreviations

BM: Brain metastases
C.I.: Confidence interval
CSF: Cerebrospinal fluid
EVD: External ventricular drainage
LM: Leptomeningeal disease
N/A: Not applicable
OR: Odds ratio
VIF: Variance inflation factor
